# Fatigue affects quality of movement more in ACL-reconstructed soccer players than in healthy soccer players

**DOI:** 10.1007/s00167-018-5149-2

**Published:** 2018-09-27

**Authors:** N. van Melick, L. van Rijn, M. W. G. Nijhuis-van der Sanden, T. J. Hoogeboom, R. E. H. van Cingel

**Affiliations:** 1 Knee Expert Center, Vijfkamplaan 8, 5624 EB Eindhoven, The Netherlands; 2 Radboud University Medical Center, Research Institute for Health Sciences, IQ Healthcare, Nijmegen, The Netherlands; 3 Praktijk voor Fysiotherapie Vossen, Echt, The Netherlands; 4 Sport Medisch Centrum Papendal, Arnhem, The Netherlands

**Keywords:** Anterior cruciate ligament reconstruction, Neuromuscular fatigue, Movement quantity, Movement quality

## Abstract

**Purpose:**

Athletes who meet return to play (RTP) criteria after anterior cruciate ligament reconstruction (ACLR) rehabilitation still have a substantially increased risk of second ACL injury. One of the contributing factors to this increased risk could be that the RTP criteria are often not tested in an ecologically valid environment and in a fatigued state. The purpose of this cross-sectional case-control study was to investigate the influence of neuromuscular fatigue on both movement quantity and quality in fully-rehabilitated soccer players after ACLR and to compare them with healthy soccer players.

**Methods:**

ACL-reconstructed soccer players (*n* = 14) and healthy soccer players (*n* = 19) participated in the study and were matched by playing level and training hours. RTP measurements were performed on the soccer field, in both a non-fatigued and fatigued state. The RTP measurements focussed on both movement quantity (hop tests) and quality [countermovement jump with a Landing Error Scoring System (LESS) score].

**Results:**

Movement quantity did not differ between ACL-reconstructed and healthy soccer players, both expressed in absolute values and the LSI-D/ND (calculated as dominant/non-dominant*100%). However, movement quality decreased more in the ACL-reconstructed soccer players in the fatigued state compared to the non-fatigued state.

**Conclusions:**

Ideally, RTP measurements should focus on movement quality and should be conducted on the soccer field in a fatigued state, creating an ecologically valid environment. The LSI-D/ND can be used as an outcome parameter for RTP measurements of movement quantity and should be at least 95%.

**Level of evidence:**

Therapeutic, Level III.

**Electronic supplementary material:**

The online version of this article (10.1007/s00167-018-5149-2) contains supplementary material, which is available to authorized users.

## Introduction

Athletes recovering from anterior cruciate ligament reconstruction (ACLR) need to be adequately evaluated during and after their rehabilitation process to ensure a safe return to play (RTP). To aid health professionals in this process, a specific set of evidence-based RTP criteria have been reported [1–3]. These criteria consist of functional performance tests based on movement quantity and quality [3, 4]. However, athletes who meet these RTP criteria still have a substantially increased risk of sustaining a second ACL injury compared with previously uninjured athletes: 10% versus 3%, respectively [5–7].

One of the contributing factors to this increased risk could be that the RTP criteria are often not tested in ecologically valid environments nor in a fatigued state, despite evidence indicating that neuromuscular fatigue is a risk factor for lower extremity injuries in healthy individuals [8–10]. There is also evidence demonstrating that neuromuscular fatigue decreases functional performance, decreases knee stability and increases tibial translation in healthy athletes and athletes after ACLR, resulting in both reduced movement quantity and quality and a probable increased risk of ACL injury [8, 11–17].

For RTP tests of movement quantity, the Limb Symmetry Index (LSI) is used as the primary outcome parameter. The LSI quantifies strength and hop performance of the operated leg as a percentage of the non-operated leg. At the end of the rehabilitation process, the LSI should be at least 90% to minimise the risk of re-injury; for pivoting athletes, strength measures should be at least 100% [18]. However, the LSI might overestimate the function of the operated knee as deficits in strength and hop performance have also been demonstrated in the non-operated leg following ACL injury [19, 20]. Consequently, although the LSI is higher than 90%, the absolute values of strength or hop tests can still be insufficient compared with preinjury values or healthy peers [19, 20]. Therefore, a new way to calculate the LSI is proposed, namely by dividing the value of the dominant leg (D) by the value of the non-dominant (ND) leg; this is called the LSI-D/ND [21, 22]. This method allows comparison between injured and healthy athletes.

Athletes who do meet the current quantitative criteria for RTP after ACLR may not do so in an ecologically valid environment and a fatigued state or might be underperforming compared to their healthy peers. Furthermore, neuromuscular fatigue might cause a deterioration of movement quality that is different in athletes after ACLR compared to healthy peers. Therefore, the aim of this study was to investigate the influence of neuromuscular fatigue on both movement quantity (absolute values and the LSI-D/ND) and quality in ACL-reconstructed soccer players and to compare them with healthy soccer players in an ecologically valid environment: the local soccer field.

The first hypothesis is that both outcome measures for movement quantity (absolute values and the LSI-D/ND) will not differ between ACL-reconstructed and healthy soccer players in a non-fatigued state. In a fatigued state, we hypothesize that the LSI-D/ND will not differ between ACL-reconstructed and healthy soccer players, while the absolute values are expected to be different. The second hypothesis is that neuromuscular fatigue will decrease movement quality more in ACL-reconstructed than in healthy soccer players. To our knowledge, this is the first study to determine the relevance of ecologically valid RTP testing (i.e. in a fatigued state at the local playing field). Testing in this way could avoid false positive RTP testing scores in ACL-reconstructed soccer players.

## Materials and methods

Recreational ACL-reconstructed soccer players and healthy recreational soccer players participated in this cross-sectional study. Two groups of healthy soccer players were chosen based on the playing level and training hours of ACL-reconstructed soccer players. ACL-reconstructed soccer players were included if their own physical therapist considered them to be fully rehabilitated based on the hop test battery of Gustavsson (LSI > 90%) [3, 23].

### Participants

Male recreational ACL-reconstructed soccer players aged between 18 and 30 years old who had ACLR in the VieCuri hospital (Venlo/Venray, the Netherlands), Bernhoven hospital (Oss/Veghel/Uden, the Netherlands) or Clinic ViaSana (Mill, the Netherlands) were invited to participate in this study at the end of their rehabilitation. Up until that point, they attended two to three physiotherapy sessions a week with an experienced sports physical therapist that worked according to ACLR practice guidelines [3]. Exclusion criteria for the ACL-reconstructed soccer players were: other injuries of the lower back, hip, knee or ankle at the moment of testing, knee effusion at the moment of testing, a contralateral ACL injury or previous ipsilateral ACLR.

The control group consisted of healthy male recreational soccer players that played soccer less than, or equal to, three times per week but did not follow a professionally designed training program [1]. Exclusion criteria for the healthy soccer players were: ACL injury or ACLR in the past, other injuries in the lower back, hip, knee, or ankle in the past 4 weeks.

All subjects provided signed, informed consent for participation in this study.

### Study procedure

Soccer players were not allowed to participate in strenuous physical activities on the day before testing and wore their own soccer footwear during the measurements, except for the vertical jump (to avoid possible damage to the contact mat). All activities were performed on the soccer field.

Before the RTP measurements in the non-fatigued state, all soccer players completed a warmup session consisting of 5 min running at an average speed of 9 km/h and 10 jumping squat repetitions with a knee angle of 90°. The Borg Rating of Perceived Exertion (RPE) scale was used to measure fatigue on a 6 to 20 scale before measurements were taken in the non-fatigued state [24]. After the initial RTP measurements, the soccer players participated in a 1-h, soccer-specific field training session. In addition to soccer specific drills, exercises focussing on speed, stability, and coordination were included in this session. After the field training, fatigue was measured again using the Borg RPE scale and RTP measurements were performed in the fatigued state.

### RTP measurements

The RTP measurements focussed on both quantitative and qualitative aspects of functional performance. All ACL-reconstructed soccer players were familiar with the RTP measurements as these were implemented during their rehabilitation. The healthy soccer players had never performed these RTP measurements previously.

All measurements were performed by the same independent examiner and physical therapist (LvR) who was not involved in the rehabilitation of the included ACL-reconstructed soccer players. The examiner was not blinded for operated/healthy status or operated leg.

### Measurements of movement quantity

The hop test battery according to Gustavsson et al. [23] was used for movement quantity measurement: a single-leg vertical jump, a single-leg hop for distance, and a single-leg side hop. This test battery has a sensitivity of 91% and the test–retest reliability of the tests is 0.89, 0.94 and 0.87 respectively [23].

For the absolute values of the hop tests (in meters or number of hops), the results of the operated leg of the soccer players after ACLR and the results of the non-dominant leg of the healthy soccer players were used for data analysis [25]. In addition to the absolute values, the LSI-D/ND was calculated and used for data-analysis [21, 22]. To determine the dominant leg for calculation of the LSI-D/ND, the question “if you were to shoot a ball at a target, which leg would you shoot with?” was used [22].

The number of athletes not meeting the RTP criterion of LSI > 90% was also calculated.

### Measurement of movement quality

A double-leg countermovement jump (CMJ) (test–retest reliability: 0.98) with frontal and sagittal plane video analyses (iPad with Hudl technique application) was used for movement quality measurement, with the first landing analysed using the Landing Error Scoring System (LESS) [26–32]. The LESS is a reliable (intra-rater ICC 0.97) measure, consisting of 17 items of landing technique errors on a range of readily observable items. A LESS score ≥ 6 indicates poor technique when landing from a jump (maximum score 19) and might increase the risk for an ACL injury [31–33]. For the LESS score, the results of the operated leg of the soccer players after ACLR and the results of the non-dominant leg of the healthy soccer players were used for data analysis. The number of athletes not meeting the RTP criterion of LESS < 6 was also calculated.

All measurements of movement quantity and quality are described in detail in the Appendix in ESM.

This study was conducted according to the ethical guidelines and principles of the international Declaration of Helsinki and was approved by the medical ethics committee of the Radboudumc Nijmegen (2017-3361).

### Statistical analyses

Sample size was calculated with G*Power, using fatigue-induced decline in functional performance in soccer players after ACLR compared with healthy controls as a primary outcome measure. Augustsson et al. [11] compared the single-leg hop performance under non-fatigued and fatigued conditions in patients after ACLR. Based on this research, the following values were used for sample size calculation: (1) mean result non-fatigued hop condition involved side 141 cm, (2) mean result fatigued hop condition involved side 109 cm, (3) standard deviation (SD) group 1: 21, (4) SD group 2: 21 [11]. An alpha of 0.05 and a power of 0.80 was used for power calculation. A sample size of 14 subjects was required.

For statistical analyses, first, to describe our study population, means and dispersion values were calculated for all soccer players’ characteristics. To compare the baseline characteristics between the ACL-reconstructed soccer players and their healthy peers, Chi-square tests and independent samples t-tests were used, where appropriate. Second, to test our hypotheses, means and dispersion values were calculated for the movement quantity and quality measurements. Repeated measures ANOVA was used to determine whether there was an effect of fatigue (i.e. non-fatigued versus fatigued) and/or group (i.e. ACLR versus healthy) on movement quantity and quality measurement results. Levene’s test was used to test equality of variances. A Chi-square test was used to calculate if there was a difference between groups in the number of athletes not meeting the RTP criterion in both the non-fatigued and fatigued state.

Data analysis was performed with IBM SPSS Statistics 22.0 (SPSS Inc., Chicago, Illinois).

## Results

Between December 2016 and July 2017, 14 soccer players at a mean time of 12.4 months after ACLR and 19 healthy soccer players were included. Characteristics of all soccer players are listed in Table 1. There were no significant differences between groups at baseline.

**Table 1:** Characteristics of the soccer players

	ACLR	Healthy	*p* value
Number of soccer players	14	19	–
Age, years (mean ± sd)	23.2 ± 3.6	21.3 ± 3.0	n.s.
Body Mass Index, kg/m^2^ (mean ± sd)	23.4 ± 1.8	21.5 ± 4.8	n.s.
Time post-surgery, months (mean ± sd)	12.4 ± 3.5	–	–
Operated leg, right (*N* [%])	7 [50]	–	–
Dominant leg, right (*N* [%])	10 [71]	15 [79]	n.s.
Training hours (mean ± sd)	3.7 ± 2.1	4.3 ± 1.5	n.s.
Borg RPE scale non-fatigued (mean ± sd)	7.3 ± 1.1	8.2 ± 2.3	n.s.
Borg RPE scale fatigued (mean ± sd)	14.9 ± 1.1	14.1 ± 2.7	n.s.

*RPE* rate of perceived exertion

For both groups, there was a significant difference (*p* < 0.001) between the Borg RPE scale in the non-fatigued and fatigued state.

### Measurement of movement quantity

Mean absolute values of the hop tests (Table 2) showed no significant before-after effect, no group effect and no time*group interaction for the vertical jump. For the hop for distance, no significant before-after effect or group effect was found but a significant time*group interaction was found (*p* = 0.042) indicating that the ACL-reconstructed soccer players jumped a shorter distance in the fatigued state (1.70 versus 1.66 m), while their healthy peers did not. For the side hop, no significant before-after effect or group effect was found but a significant time*group interaction (*p* = 0.022) was reported, indicating that the number of hops for the ACL-reconstructed soccer players decreased in the fatigued state (59 versus 56 hops) while the number of hops for their healthy peers did not.

**Table 2:** Mean absolute values, limb symmetry indices (LSI) and number of athletes not meeting the RTP criterion of LSI > 90% of the hop test battery of Gustavsson (movement quantity) in the non-fatigued and fatigued state

	ACLR N-F	ACLR F	Healthy N-F	Healthy F
Vertical jump, absolute value in m (mean ± sd)	0.24 ± 0.05	0.23 ± 0.05	0.23 ± 0.06	0.24 ± 0.06
Vertical jump, LSI-D/ND in % (mean ± sd)	100.1 ± 15.4	99.1 ± 19.7	97.7 ± 12.5	97.6 ± 11.4
Athletes not meeting the LSI > 90% criterion, *N* (%)	2 (14)	3 (21)	8 (42)	6 (32)
Hop for distance, absolute value in m (mean ± sd)	1.70 ± 0.18^a^	1.66 ± 0.18	1.61 ± 0.28	1.66 ± 0.28
Hop for distance, LSI-D/ND in % (mean ± sd)	97.2 ± 5.5	95.6 ± 8.0	96.8 ± 8.4	96.3 ± 8.0
Athletes not meeting the LSI > 90% criterion, *N* (%)	2 (14)	4 (29)	6 (32)	4 (21)
Side hop, absolute value in *N* (mean ± sd)	59 ± 11^a^	56 ± 12	55 ± 12	57 ± 10
Side hop, LSI-D/ND in % (mean ± sd)	95.8 ± 10.6	95.1 ± 14.3	100.0 ± 17.9	99.7 ± 9.6
Athletes not meeting the LSI > 90% criterion, *N* (%)	4 (29)	3 (21)	4 (21)	4 (21)

*N-F* non-fatigued, *F* fatigued

^a^Significant difference between non-fatigued and fatigued state

For the LSI-D/ND (Table 2), no significant effects were found for the vertical jump, hop for distance or side hop.

In the non-fatigued state, there were two (vertical jump and hop for distance) to four (side hop) ACL-reconstructed soccer players that did not meet the RTP criterion for LSI > 90%, despite their own physical therapist reporting that they had met this criterion. However, there were no significant differences between the number of athletes not meeting the RTP criterion when comparing ACL-reconstructed with healthy soccer players, neither in the non-fatigued state nor in the fatigued state (Table 2).

### Measurement of movement quality

**Fig. 1:**
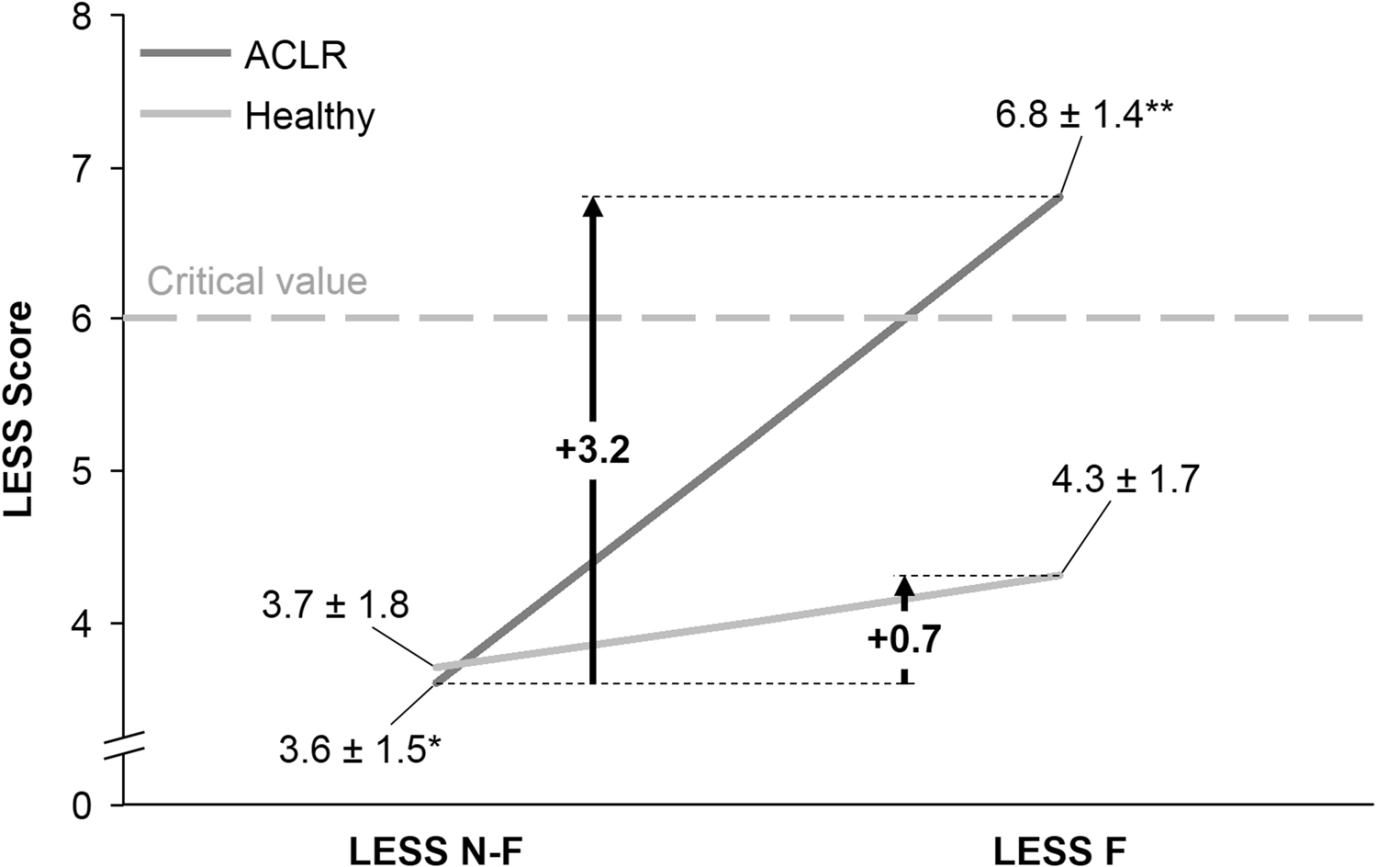
Landing Error Scoring System (LESS) score for ACL-reconstructed and healthy soccer players in the nonfatigued and fatigued state

LESS scores increased significantly in the fatigued state (*p* < 0.001), were significantly higher in the ACL-reconstructed soccer players (*p* = 0.026), and increased significantly more in the ACL-reconstructed soccer players compared to their healthy peers (*p* < 0.001) (Table 3; Fig. 1).

**Table 3:** Landing Error Scoring System (LESS) score at the double-leg countermovement jump (movement quality) in the non-fatigued and fatigued state

	ACLR N-F	ACLR F	Healthy N-F	Healthy F
LESS, score (mean ± sd)	4 ± 2^a^	7 ± 1^b^	4 ± 2	4 ± 2
Athletes not meeting the LESS < 6 criterion, *N* (%)	2 (14)	12 (86)^b^	2 (11)	6 (32)

*N-F* non-fatigued, *F* fatigued

^a^Significant difference between non-fatigued and fatigued state

^b^Significant difference with healthy soccer players

In the non-fatigued state two athletes in both groups were not able to meet the RTP criterion of LESS < 6. However, in the fatigued state there was a significant difference between groups (*p* = 0.002), with 12 (86%) of the ACL-reconstructed soccer players not meeting the criterion compared to six (32%) of the healthy soccer players (Table 3).

## Discussion

The most important finding of the present study was that, in a fatigued state, the LESS score increased more in the ACL-reconstructed soccer players compared to healthy soccer players, when tested on the soccer field (ecologically valid environment). Moreover, the number of athletes not meeting the LESS RTP criterion increased drastically in the fatigued state. Movement quantity (both absolute values and LSI-D/ND) did not differ between ACL-reconstructed and healthy soccer players. These findings suggest that movement quality measurement in a fatigued state should be used in RTP testing of ACL-reconstructed soccer players.

For movement quantity measurements, the first hypothesis that both outcome measures (absolute values and the LSI-D/ND) would not be different between ACL-reconstructed soccer players and their healthy peers in a non-fatigued state was confirmed. However, in the fatigued state there was also no difference in both absolute values and the LSI-D/ND between ACL-reconstructed and healthy soccer players, while it was expected that absolute values would be different. Nevertheless, the ACL-reconstructed soccer players had a significantly decreased performance when comparing the non-fatigued with the fatigued state. The first hypothesis was based on the findings of Wellsandt et al. [20] and Gokeler et al. [19] who found that ACLR patients were able to reach the LSI cut-off of 90% at 6 and 7 months, respectively, without reaching their estimated pre-injury capacity or the normative values of healthy controls. It is unclear whether the ACLR patients in the study of Wellsandt et al. [20] and Gokeler et al. [19] had completed their rehabilitation. The aforementioned results imply that they had not, which is also supported by the findings that ACLR rehabilitation should last at least 9 months to minimise the risk for a second ACL injury [34]. An alarmingly high number of pivoting athletes (72–86%) are released to full, unrestricted sports activities without meeting the RTP criteria, despite a plethora of evidence showing that not meeting the quantitative RTP criteria increases the risk for a second ACL injury [5, 34–36].

The LSI-D/ND, which compares the values of the dominant and non-dominant leg, showed no differences between ACL-reconstructed and healthy soccer players, both in a non-fatigued and fatigued state. Therefore, using the LSI-D/ND could be useful in RTP measurements of pivoting athletes after ACLR. Normally, a cut-off value of 90% is sought [5, 18, 34] but the results in this study (see Table 2) suggest that the LSI-D/ND should be at least 95% for hop tests at the end of ACLR rehabilitation.

For movement quality measurement, the LESS was significantly higher in the ACL-reconstructed soccer players than in their healthy peers (6.8 and 4.3, respectively) in a fatigued state, but not in the non-fatigued state (3.6 and 3.7, respectively). Gokeler et al. [14] found different results in their study when comparing the LESS between patients 10 months after ACLR and healthy controls in a non-fatigued and fatigued state. They used a fatigue protocol of 10 double-legged squats to 90 degrees of knee flexion and 2 double-legged CMJ’s, which were repeated until it was no longer possible to reach 70% of the maximum CMJ height for 2 trials [14]. They also used an RPE score to rate fatigue, however, their fatigued state RPE of 18.7 was higher than the 14.1 (healthy soccer players) and 14.9 (soccer players after ACLR) in this study. Gokeler et al. [14] found that ACLR patients already had a higher LESS in the non-fatigued state (6.5 versus 2.5). In a fatigued state, the difference was smaller: 7.0 for ACLR patients and 6.0 for healthy controls. It is unclear if these patients had finished their rehabilitation but considering two patients did not feel confident enough to perform the LESS protocol in the fatigued state, it would suggest that they were not fully rehabilitated [14]. The ACLR patients in this study were 12.4 months postoperative and had completed their rehabilitation, which might explain the difference in the non-fatigued LESS with the ACLR patients of Gokeler et al. [14].

The majority of ACL-reconstructed soccer players in this study might still be at risk for a second ACL injury, because it was found that, on the soccer field, four soccer players did not meet at least one RTP criterion in the non-fatigued state. Interestingly, all soccer players met the hop test RTP criterion of LSI > 90% in the physical therapy practice before partaking in the study. Apparently, the different conditions (e.g. surface and wind) of this ecologically more valid environment appear to make it more difficult to meet the hop test RTP criterion. Moreover, considering that soccer players with a LESS ≥ 6 are suspected to be more prone to a first-time ACL injury (which also might hold true for the risk for a second ACL injury), 86% of the ACL-reconstructed soccer players and 32% of their healthy peers in our study, had an increased risk [37].

This study has some limitations. First, the use of a soccer-specific training in this study, with a BORG scale as the measurement of fatigue, could cause a different form of fatigue than fatigue protocols used in other studies. However, the training produced soccer-specific fatigue, implying an ecologically valid fatigue protocol used in this study. Second, all ACL-reconstructed soccer players were familiar with the RTP measurements as these were implemented during their rehabilitation, but the healthy soccer players had never performed these RTP measurements before. Healthy soccer players could have had a learning effect in movement quantity, visible in the absolute values of all hop tests increasing in the fatigued state compared to the non-fatigued state. However, this still implies that the ACL-reconstructed soccer players have an acceptable performance compared to their healthy peers.

The results of the present study can be used in day-to-day clinical practice when rehabilitating ACL-reconstructed soccer players. Determining the moment for RTP based on hop tests and movement quality measurement performed in the physical therapy practice could cause false positive RTP scores, allowing soccer players to return to play too early, with a possible increased risk for a second ACL injury. According to the results of the present study, when testing on the soccer field and in a fatigued state (ecologically valid environment), prolonged rehabilitation seems necessary to meet the RTP criteria for movement quality and have comparable results to healthy peers.

## Conclusions

For ACL-reconstructed soccer players, the LSI-D/ND should ideally be at least 95% for hop tests at the end of rehabilitation. Moreover, hop tests and movement quality measurement are suggested to be performed in an ecologically valid environment (i.e. in a fatigued state at the local playing field) to avoid false positive RTP testing scores in ACL-reconstructed soccer players.

## Electronic supplementary material

Below is the link to the electronic supplementary material.

Supplementary material 1 (DOCX 194 KB)

Supplementary material 2 (SAV 5 KB)
