# Supplementary material for: Fatigue affects quality of movement more in ACL-reconstructed soccer players than in healthy soccer players
Source: Knee Surg Sports Traumatol Arthrosc. 2018 Sep 27;27(2):549–55. doi: 10.1007/s00167-018-5149-2 (PMC6394549; doi:10.1007/s00167-018-5149-2)
Supplement: Supplementary file 1 — Supplementary material 1 (DOCX 194 KB) [file 167_2018_5149_MOESM1_ESM.docx]

**APPENDIX: MEASUREMENTS OF FUNCTIONAL PERFORMANCE**

| **Test** | **Subject’s start position** | **Procedure** | **Sessions** | **Recorded variable** | **Picture*** |
| --- | --- | --- | --- | --- | --- |
| **Quantity of movement** | | | | | |
| Vertical jump | Upright position, standing on one leg with the hands placed behind the back [16]. | The subject quickly bents his knee as much as desired and then immediately jumped upwards, attempting to maximise the height jumped. The subject had to perform a controlled, balanced landing and had to keep the landing foot in place (2–3 s) [16]. A Pro-Jump contact mat (ProCare, the Netherlands) was used to measure jump height in centimeters. This test was performed on football socks, because of the chance of damage to the contact mat when wearing soccer footwear. | Two practice sessions, before three test sessions for each leg. Between practice and test sessions, a one minute rest period. Between each test session, a 30 second rest period. The non-operated leg was tested before the operated leg. | The highest jump of the three test sessions in meter.  The LSI-D/ND was calculated as the value of the dominant leg divided by the value of the non-dominant leg multiplied by 100. | 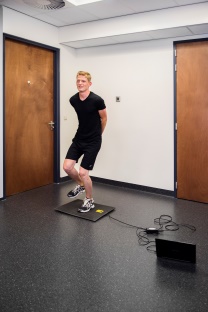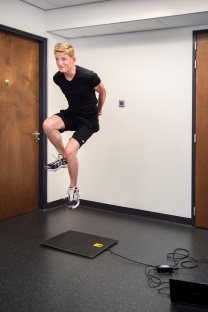 |
| Hop for distance | Upright position, standing on one leg with the hands placed behind the back [16]. | The subject hopped as far  as possible and landed on the same leg. Free leg swing was allowed. The subject had to perform a controlled, balanced landing and had to keep the landing foot in place until (2–3 s) the test  leader had registered the landing position. Failure to do so resulted in a disqualified hop. The distance was measured in centimetres from the toe at the push-off to the heel where the subject landed [16]. | Two practice sessions, before three test sessions for each leg. Between practice and test sessions, a one minute rest period. Between each test session, a 30 second rest period. The non-operated leg was tested before the operated leg. | The furthest jump of the three test sessions in meter.    The LSI-D/ND was calculated as the value of the dominant leg divided by the value of the non-dominant leg multiplied by 100. | 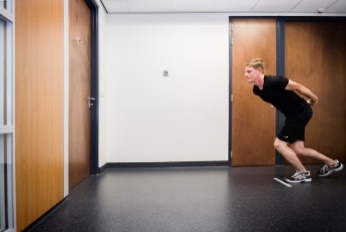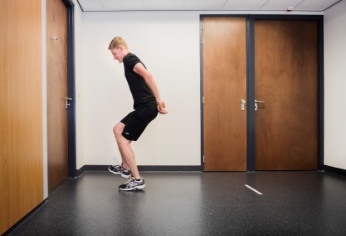 |
| Side hop | Upright position, standing on one leg with the hands placed behind the back [16]. | The subject jumped from side to side between two parallel lines. The lines were created by foam (Kadeem marker spray) placed 40 cm apart on the soccer field. The subjects were instructed to jump as many times as possible during a period of 30 s. The number of successful jumps performed, without touching  the tape, was recorded. Touching the tape was recorded as an error [16]. | A few practice jumps were allowed to familiarise themselves with the jumping distance, before they performed one test session of 30 seconds. The non-operated leg was tested before the operated leg. | The amount (N) of correct jumps.  The LSI-D/ND was calculated as the value of the dominant leg divided by the value of the non-dominant leg multiplied by 100. | 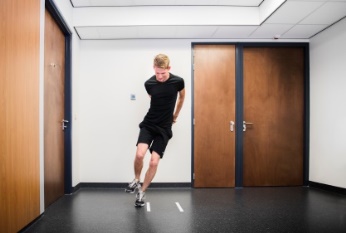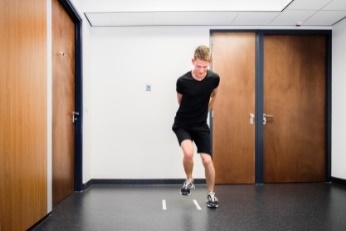 |
| **Qualitaty of movement** | | | | | |
| Double-leg countermovement jump | The subject started at two legs with the feet hip width apart. | Subjects had to jump as high as possible, perform a landing on two feet and immediately jump as high as possible again. Arm swing was allowed during the jumps [27,28,30]. | One practice session was allowed before two test sessions. One session was frontally filmed, one session was sagittally filmed at the side of the operated leg (soccer players after ACLR) and non-dominant leg (healthy soccer players) [24]. | The first landing of the countermovement jump was used to analyse with the Landing Error Scoring System (LESS). | 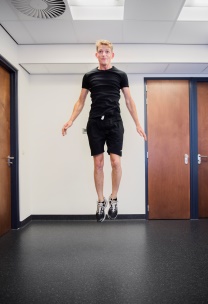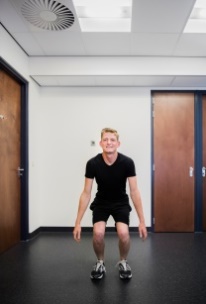 |

*The pictures are examples of the tests in a clinical setting. All tests in this study were performed on the soccer field.
